# Sociodemographic Factors as Predictors of the Duration of Long-term Psychotherapy: Evidence from a Finnish Nationwide Register Study

**DOI:** 10.1007/s10488-023-01305-7

**Published:** 2023-10-12

**Authors:** Sanna Selinheimo, Kia Gluschkoff, Johanna Kausto, Jarno Turunen, Ari Väänänen

**Affiliations:** Finnish Institute of Occupational Health, Helsinki, Finland

**Keywords:** Psychotherapy duration, Socioeconomic factors, Gender, Access to health care, Mental health services

## Abstract

**Objective:**

The role of sociodemographic factors in determining psychotherapy duration has been largely unexplored despite their known association with treatment use. We examined the association between sociodemographic factors and rehabilitative psychotherapy treatment duration, as well as any changes in duration over time.

**Method:**

We used three register-based nationally representative cohorts. Participants included employed Finnish individuals (n = 5572, 77% women, mean age = 37) who started psychotherapy treatment in 2011, 2013 or 2016 and were followed until 2019. We used negative binomial regression to examine the association between sociodemographic factors (age, gender, education, occupational status, income, geographical area of residence, and onset year of treatment) with treatment duration.

**Results:**

The mean treatment duration was 27 months (with a standard deviation of 12 months). Several sociodemographic factors were associated with treatment duration. Gender and education were found to have the largest impact on treatment duration, with females having a longer duration (IRR 1.08, 95% CI 1.04–1.11) and those with low education having a shorter duration (IRR 0.91, 95% CI 0.85–0.97), resulting in a difference of 2–3 months. Treatment duration also increased in later years, which suggests potentially increasing differences in treatment implementation. At largest, the combined effect of all factors corresponded to a 10-month difference in treatment duration.

**Conclusions:**

The duration of long-term psychotherapy varied across the sociodemographic groups and increased in all studied groups in the 2010s.

**Supplementary Information:**

The online version contains supplementary material available at 10.1007/s10488-023-01305-7.

## Objective

Mental health care equality entails equitable access to treatment, its contents, and duration based on the patient’s needs, rather than contextual factors linked to their sociodemographic background. However, treatment duration and termination can be affected by various factors, including the quality of the therapist-client alliance, therapist effects, and client clinical characteristics. Often, these factors are examined while neglecting the impact of sociodemographic factors that can affect a client’s capacity to engage in psychotherapy.

Research has demonstrated that a poor treatment alliance is associated with early termination of therapy. Patients’ decisions to leave treatment prematurely have been attributed to less clarifying treatment experiences and a lack of perceived improvement (Kegel & Flückiger, 2015). Meta-analytic evidence has also confirmed that a weaker therapeutic alliance is associated with early treatment termination (Sharf et al., 2010). However, in their meta-analysis already in 1993, Wierzbicki and Pekarik discovered that clients with low education, low socioeconomic status or who belong to an ethnic minority, were more likely to end treatment beneficial to their clinical condition (Wierzbicki & Pekarik, 1993). These demographic factors have not consistently predicted treatment termination in a more recent meta-analysis, which may be due to the infrequent reporting of such factors in original study reports (Swift & Greenberg, 2012). Nonetheless, higher education has been identified as a factor that helps sustain treatment continuity despite alliance ruptures, as noted in a study by Sharf et al. (Sharf et al., 2010).

Studies exploring factors influencing treatment duration have revealed similar findings. The client´s suitability for psychotherapy has been associated with varying needs for treatment duration (Alanne et al., 2021; Ingenhoven et al., 2012; Laaksonen et al., 2013). Additionally, treatment-related factors such as therapist effects (Lutz et al., 2015) have been linked with differences in treatment duration. However, evidence also exists regarding contextual factors, such as the client’s education (Rabinowitz & Renert, 1997), financial situation, number of sessions approved by the social security system or insurance companies (Lutz et al., 2015; Thompson et al., 2018) and practical access barriers to the treatment (e.g. transportation possibilities and schedules) (Slaunwhite, 2015) that predict psychotherapy treatment duration. Furthermore, clients’ financial distress and lack of structural possibilities to choose a therapist or influence treatment duration have been linked to a higher likelihood of early withdrawal and worse treatment outcomes (Seligman, 1995; Thompson et al., 2018).

Studies on factors influencing psychotherapy treatment duration are largely based on small-scale naturalistic studies in selected patient populations or randomized controlled trials. Research has typically examined the association between either treatment duration and treatment outcomes (Bone et al., 2021; Stulz et al., 2013) or socioeconomic factors or area of residence and activity to use the treatment (Delgadillo et al., 2016; Evans-Lacko et al., 2018). However, there is a lack of research linking socioeconomic factors, area of residence, and treatment duration. Randomized controlled trials are limited in assessing this issue as they have fixed treatment protocols with a predetermined number of sessions and patients selected based on clinical criteria rather than socioeconomic criteria. However, sociodemographic factors and area of residence have been found to influence activity in seeking mental health treatment (Finegan et al., 2020; Meadows et al., 2015; Niemeyer & Knaevelsrud, 2022; Packness et al., 2017). Therefore, focusing on socioeconomic factors as predictors of treatment duration may reveal systematic differences in treatment implementation and withdrawal from treatment among different population groups, which could be important in improving treatment efficacy and reducing barriers to accessing treatment.

In Finland, rehabilitative psychotherapy is the primary publicly provided form of rehabilitation for individuals at risk of disability in work or study due to mental disorders. It is partly subsidized by the social security system^1^ and is granted for a maximum of 80 sessions per year and 200 sessions per three years, including various therapeutic approaches (i.e., cognitive, cognitive-behavioural, cognitive analytic, psychodynamic, integrative, solution-focused or family therapy). To be eligible for therapy, individuals must be between 16 and 67 years old and at risk of disability due to mental disorders. Since 2011, it has been granted statutorily to all at-risk individuals, increasing annual users from 18 245 in 2011 to 50 392 in 2019 (Social Insurance Institution, 2022). Despite its statutory status, psychotherapy is not easily accessible as patients require a referral from a psychiatrist. A minimum of three-month follow-up is required to assess whether first-line treatments (primarily pharmacological, in some cases short-term counselling or internet-delivered therapy) are effective for improving functioning is required before referral. Evidence suggests that treatment may reduce work disability at the population level (Kausto et al., 2022) but its use has been found to be modified by sociodemographic factors (Leppänen et al., 2022). In addition, despite the improvement in treatment coverage with its statutory status, regional inequalities in treatment provision exist, with services being concentrated in university hospital areas with high population density (Patana, 2014).

To conclude, the vast majority of studies have not reported socioeconomic factors of study completers and drop-outs in psychotherapy research (Cooper & Conklin, 2015; Swift & Greenberg, 2012), which makes it challenging to evaluate whether socioeconomic factors influence treatment implementation. As a result, the role of sociodemographic-level characteristics in determining the treatment duration remains largely unexamined despite their importance in achieving and engaging the treatment. Due to the costs and time requirements of treatment, factors related to occupational characteristics, such as the ability to attend treatment regularly and area of residence, may influence the possibility of engaging in and continuing psychotherapy. Therefore, in this study, we aimed to examine whether socioeconomic factors or area of residence are associated with differences in treatment duration.

## Method

### Study Population

#### Sample

We used data from the Rise of Mental Vulnerability project (Kausto et al., 2022; Olakivi et al., 2023), which drew cohorts of 33% random samples from the working-age population (18–64 years) in 2010 (N = 1,115,832), 2013 (N = 1,105,519) and 2016 (N = 1,093,429) censuses in the Statistics Finland population database. In this study, we selected those who started rehabilitative psychotherapy in 2011 for the first cohort, and in 2013 or 2016 for the following cohorts. The cohorts were followed from their baseline (2011, 2013 or 2016) until 2019 (i.e. for 4 to 9 years, depending on their baseline). We only included employed individuals in the study to examine the association between occupational status and treatment duration.

#### Measures

***Rehabilitative psychotherapy.*** The primary outcome variable was the individual duration of rehabilitative psychotherapy in months. The data for this study was obtained from the Social Insurance Institution of Finland, where rehabilitative psychotherapy usage is registered based on reimbursement dates. To approximate the total treatment duration, we subtracted the first day of psychotherapy reimbursement from the last day. This calculation included breaks within treatment periods (such as holidays) and between periods (between the first and second, and between the second and third). After five years of completing the previous treatment, it is possible to have a new maximum three-year set of rehabilitative psychotherapy. However, in this study, only one set of psychotherapy treatments, with a maximum of three periods, was included per individual. As a sensitivity analysis, we also calculated the total treatment duration by determining the duration of each period according to its first and last reimbursement date and summing the resulting durations. This method excluded potential breaks in treatment between the periods. In this study, we included only those individuals whose psychotherapy had begun after it became statutory in 2011.

***Sociodemographic characteristics.*** Independent variables were age (divided by 10 in the regression analysis to assist the interpretation of results), gender (male, female), education, occupational status, income (in quartiles), and area of residence. Education was categorized as high, medium, or low. The occupational grade was coded as upper-level employees (those with administrative, managerial, professional and related occupations), lower-level employees (those with administrative and clerical occupations) and manual workers (workers in agriculture or similar, manufacturing, other production and distribution and service workers, workers unspecified) (ILO, 2010). Individual´s income was measured as the total annual taxable gross income and the participants were grouped into quartiles based on the income distribution in the cohorts. This approach accounts for the participants’ relative income position, minimizing the influence of inflation and changes in wage levels. Area of residence was determined using four out of five of the Nomenclature of Territorial Units for Statistics areas (level 2), which include Capital area (Helsinki and Uusimaa), Southern Finland, Western Finland, and Northern/Eastern Finland (European Commission, 2020), excluding Åland, an autonomous area of Finland. Sociodemographic factors were measured in the year rehabilitative psychotherapy began (in 2011 for the 2010 cohort and in 2013 or 2016 for the more recent cohorts).

Data on sociodemographic characteristics for each cohort were obtained from Statistics Finland, while information on psychotherapy use was collected from the Social Insurance Institution of Finland. National ID numbers, which are unique to each permanent resident in Finland, were used to link data on psychotherapy use to the participants’ sociodemographic characteristics. All data were anonymized before being made available to the researchers.

#### Statistical Methods

We first calculated descriptive statistics for the baseline characteristics of each cohort. To examine differences between the cohorts, ANOVA was used for continuous variables and Pearson’s Chi-squared test was used for categorical variables. Second, negative binomial regression was performed to determine the combined effects of age, gender, education, occupational grade, income, area of residence, and treatment onset year on psychotherapy duration. Negative binomial regression is typically used when the dependent variable is a count variable and the Poisson model assumption of equal mean and variance is not met, indicating overdispersion (Hilbe, 2011). We chose negative binomial regression after detecting significant overdispersion using the R package “performance” (Lüdecke et al., 2021). Interactions between each independent variable and treatment onset year were also tested to determine if the effects of age, gender, education, income, occupational grade, and area of residence on psychotherapy duration had changed over time. The interactions were tested using a single regression model that encompassed the main effects of all sociodemographic factors and their interaction with a categorical variable for the onset year. We present the regression results as incidence rate ratios (IRR) with 95% confidence intervals and as model-predicted marginal means for different levels of the predictors. The analyses were performed using R version 4.2.2 (R Core Team, 2021) and the results were visualized using the ggplot2 package (Wickham, 2016).

## Results

**Fig. 1:**
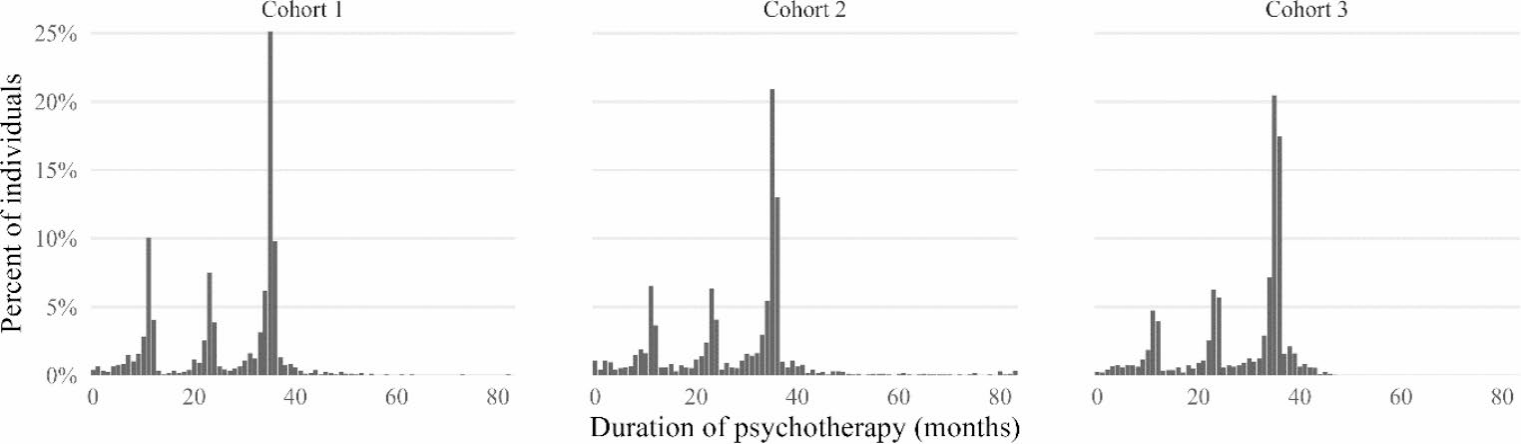
Treatment duration in months (including breaks) in study cohorts

In the 2011 cohort, 1 547 (0.22%) of 692 533 employed individuals, in 2013 1 665 (0.24%) of 684 365 employed individuals and 2016 2 359 (0.35%) of 665 766 employed individuals started psychotherapy (Table 1.). There were no significant age, gender, occupational grade, or area of residence differences between the cohorts. However, individuals in the most recent 2016 cohort had a slightly higher level of education and income than the previous cohorts. Psychotherapy duration was measured both in terms of psychotherapy duration in months and a number of psychotherapy periods was longer among those in later cohorts compared to those in the earliest cohort. The treatment durations in three cohorts are shown in Fig. 1. Overall, the mean treatment duration was 27.47 months (standard deviation 11.62 months).

**Table 1:** Demographics of the study population by cohorts

	Cohort 2010 N = 1 547	Cohort 2013 N = 1 665	Cohort 2016 N = 2 360	*p-value*
Age	37.02 (10.62)	36.56 (10.52)	37.34 (10.35)	0.064
Gender				0.4
Male	362 (23.40%)	357 (21.44%)	536 (22.71%)	
Female	1 185 (76.60%)	1 308 (78.56%)	1 824 (77.29%)	
Education				< 0.001
High	923 (59.66%)	990 (59.46%)	1 512 (64.07%)	
Intermediate	536 (34.65%)	584 (35.08%)	779 (33.01%)	
Low	88 (5.69%)	91 (5.47%)	69 (2.92%)	
Occupational status				0.9
Upper-level employees	579 (37.43%)	610 (36.64%)	866 (36.69%)	
Lower-level employees	757 (48.93%)	820 (49.25%)	1 146 (48.56%)	
Manual workers	211 (13.64%)	235 (14.11%)	348 (14.75%)	
Income	31 810 (17 200)	32 607 (18 074)	34 335 (17 626)	< 0.001
Area of residence				> 0.9
Capital area (Helsinki and Uusimaa)	628 (40.59%)	663 (39.82%)	955 (40.47%)	
South	251 (16.22%)	278 (16.70%)	392 (16.61%)	
West	374 (24.18%)	392 (23.54%)	559 (23.69%)	
East and North	294 (19.00%)	332 (19.94%)	454 (19.24%)	
Period 1. users	1 547 (100.00%)	1 665 (100.00%)	2 360 (100.00%)	
Period 2. users	1 128 (72.92%)	1 294 (77.72%)	1 938 (82.12%)	< 0.001
Period 3. users	846 (54.69%)	951 (57.12%)	1 433 (60.72%)	< 0.001
Duration including breaks	26.44 (11.56)	27.60 (13.22)	28.06 (10.36)	< 0.001
Duration excluding breaks	20.92 (8.82)	24.77 (10.77)	26.18 (9.58)	< 0.001
1 Mean (SD); n (%) 2 One-way ANOVA; Pearson’s Chi-squared test Duration excluding breaks was used as an outcome in the sensitivity analysis.				

**Fig. 2:**
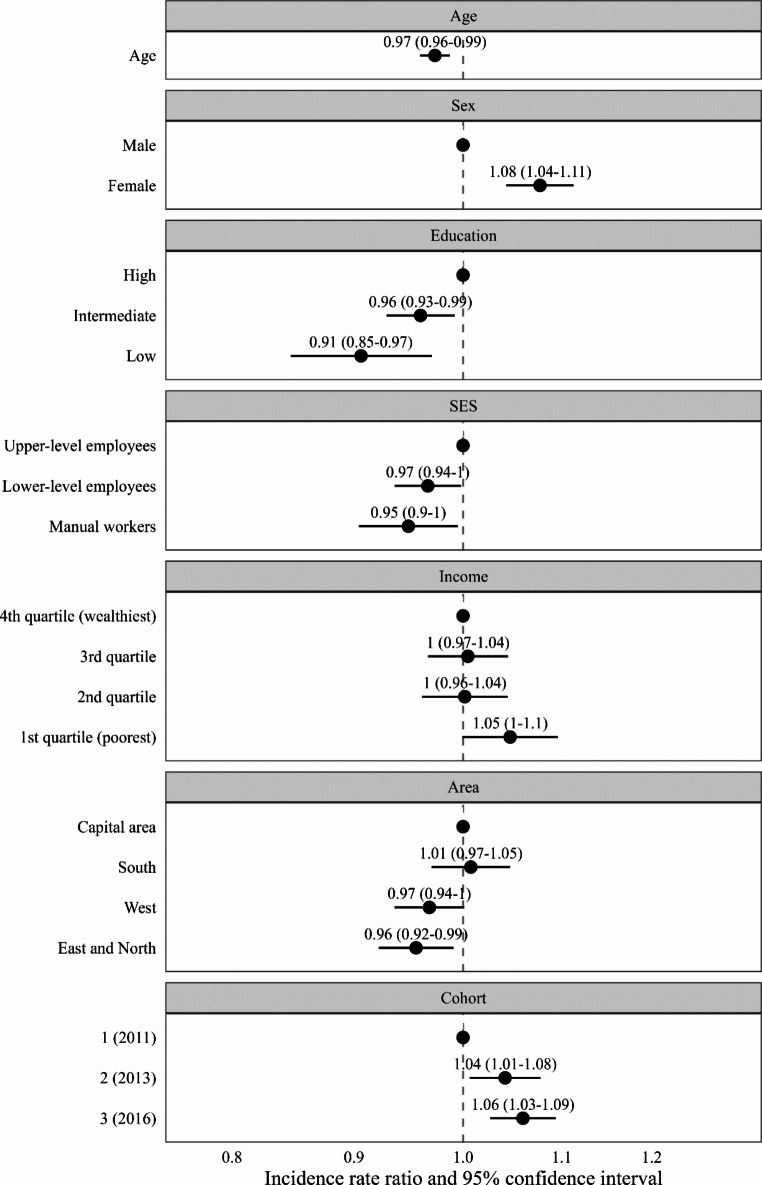
Incidence rate ratios (95% confidence interval) for the associations between sociodemographic factors and psychotherapy duration. Age = The effect of a 10-year increase in age. The total treatment duration was calculated by including breaks between treatment periods in the duration

Figure 2. shows negative binomial regression results (IRR with 95% CI) for the associations between sociodemographic factors and psychotherapy duration. For each categorical variable, one level was chosen as the reference category, and the coefficients for the other levels were interpreted in relation to this reference category. Younger age, female gender, and more recent treatment onset year were associated with longer treatment duration, while lower education, lower occupational grade and living in the sparsely populated Eastern and Northern areas were associated with shorter treatment duration. Although the poorest income quartile had a longer treatment duration than the wealthiest quartile, the differences between income quartiles were statistically nonsignificant (p = 0.052). Gender and education showed the strongest association with treatment duration, with an average difference of 2–3 months between males vs. females and low vs. high education. A 10-year increase in age was associated with less than a month shorter treatment duration. Lower occupational status and living in the sparsely populated Eastern and Northern area (vs. The Capital area) were associated with around 1-month shorter treatment duration. In addition, treatment duration was around 1 month longer in more recent cohorts.

**Fig. 3:**
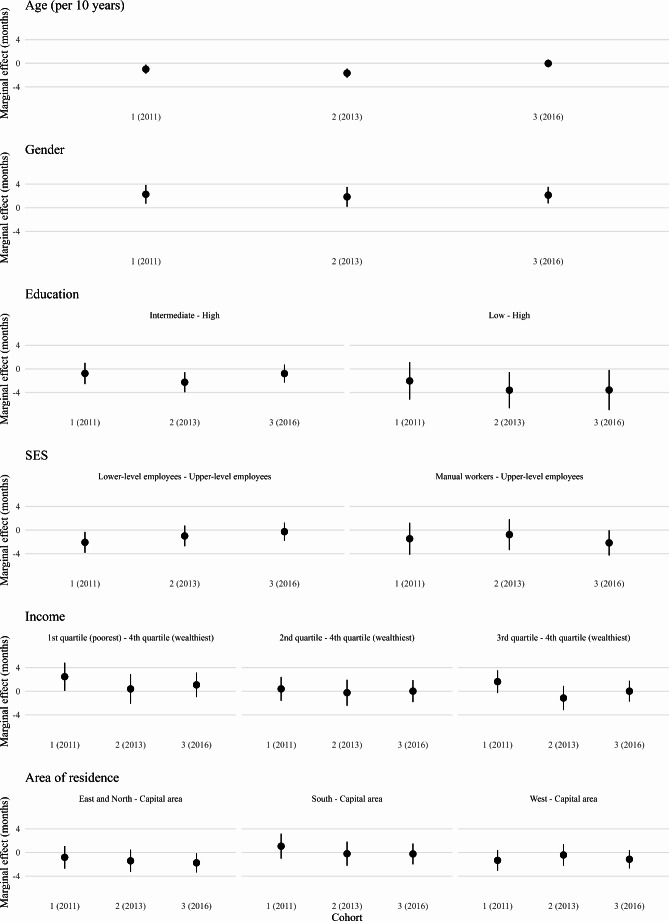
Marginal effects for predictors of treatment duration in months calculated by including breaks between treatment periods in the duration

As for the results regarding whether the associations had changed over time (i.e., potential interactions between treatment onset year and various sociodemographic factors), we did not observe any significant interactions. Nonetheless, to avoid understating potential interaction effects by basing our conclusions only on the statistical significance of the interaction terms, as recommended by literature (Berry et al., 2012; Kingsley et al., 2017), we additionally calculated and plotted marginal effects for different values of the variables (Fig. 3.). Marginal effects show how the treatment duration changes when a specific independent variable changes, holding other variables constant. According to the marginal effects, the association of age with treatment duration was present in the earlier, but no longer in the most recent cohort. The association of area of residence with treatment duration was present only in the most recent cohort. Similar conclusions were drawn when the treatment onset year was modelled as a continuous variable (results available upon request).

Based on the results of the regression model with the main effects of the predictors, we calculated the predicted treatment duration among males and females by age (30 vs. 50), educational grade (high vs. low), occupational status (upper-level employee vs. manual worker), income quartile (4th quartile (wealthiest) vs. 1st quartile (poorest)) and geographical area (Capital area vs. more sparsely populated Eastern and Northers area) for the most recent cohort (2016). It should be noted that such combinations of sociodemographic factors are rare, and the results represent the most extreme differences in treatment duration. The difference in treatment duration was at largest around 10 months: for a 50-year-old male manual worker in the wealthiest income quartile, living in Eastern or Northern Finland, the predicted treatment duration was 21.9 months (95% CI 20.9 to 23.0), whereas, for a 30-year-old upper-level female employee in the poorest income quartile, living in the capital area, the predicted treatment duration was 31.8 months (95% CI 30.1 to 33.5). The differences were of similar magnitude in the earlier cohorts (at largest 9.3 months in the 2011, and 9.7 months in the 2013 cohort). The full details of all possible combinations are shown in Supplementary Table 1.-3.

As a sensitivity analysis, we repeated the analysis by using a measure for treatment duration in which potential breaks in treatment between treatment periods were omitted from the total duration (see Supplemental Fig. 1.). The results were practically similar, except for the association between treatment onset year and treatment duration (see Supplemental Fig. 2. and 3.). When potential breaks between treatment periods were omitted from the total duration, in comparison to the 2011 cohort, treatment duration was from 3 to 5 months longer the in the following 2013 and 2016 cohorts. This difference between the main and sensitivity analysis likely results from the fact that the follow-up for the more recent cohorts was shorter than for the earliest cohort. For example, for the most recent cohort of 2016, the follow-up was 4 years, which meant that treatment durations exceeding 4 years could not be observed. Overall, it appears that treatment duration may have increased to a greater degree in the 2010s than what was estimated in the main analysis. As a second sensitivity analysis, we also assessed the association of sociodemographic factors with treatment duration in years separately for the 2011 cohort and cohorts 2013 and 2016 (see Supplemental Fig. 4.). In contrast to the results of our main analysis, in the 2011 cohort, low income was associated with longer (3-year) treatment duration.

## Discussion

The provision of psychotherapy should be equitable and based on individual needs. Results of this large and representative population-based study however suggest that sociodemographic factors are associated with differences in treatment duration in Finland during 2010s. Specifically, female gender and more recent treatment onset year were associated with longer treatment duration and lower education, lower occupational grade and living in the sparsely populated Eastern and Northern areas were associated with shorter treatment duration. Gender and education had the strongest associations with treatment duration, and the combined effect of all factors corresponded to a 10-month difference between the shortest and longest treatment duration. This indicates that the provision of psychotherapy varied systematically based on people´s demographic backgrounds. An average treatment duration increased from early to late 2010s, even though the criteria for rehabilitative psychotherapy remained the same during the study period.

Studies have consistently shown that women hold more positive attitudes towards mental illness and help-seeking for mental health issues (Ewalds-Kvist et al., 2013; Mackenzie et al., 2006; Nam et al., 2010) and psychotherapy use (Eggenberger et al., 2021) than men. Our results add to previous findings stating that the female gender is associated also with a longer treatment duration than the male gender. This finding suggests that attitudes towards mental health treatment associated with overall treatment implementation. Some evidence suggests that men, particularly those who adhere strongly to traditional masculine norms, may only seek psychotherapy when their symptoms are severe (Eggenberger et al., 2021). This tendency to seek help only when symptoms are severe may be related to men ending treatment once their symptoms alleviate even a little, while women may be more likely to continue treatment even if their symptoms improve. Furthermore, higher age is associated with shorter treatment duration but only to a very minor extent. Previous research has shown that psychotherapy use tends to decline among individuals over 40 years old (Packness et al., 2017) and particularly among the elderly (Jokela et al., 2013a; Wei et al., 2005) despite the evidence suggesting, that older adults have more positive attitudes towards seeking mental health help than younger adults (Mackenzie et al., 2006). Our results provide some indication that ageing is not only associated with decreased treatment use but also with shorter treatment duration. The moderate effects seen in our study could be explained by the age range in our study focus as the decreased treatment use has shown to be especially pronounced among the elderly (Jokela et al., 2013b; Wei et al., 2005) and should be interpreted with caution as the effect could be seen only in the earliest cohort. However, it is possible that factors such as career phase could influence the readiness and motivation to engage in long-term psychotherapy aimed at improving workability, which could explain the differences in treatment duration between younger and older adults. Therefore, future studies should comprehensively examine factors related to long-term treatment engagement across different age groups.

Taken together, our results suggest that individuals with lower levels of education, and to a lesser extent, lower occupational status, tend to have shorter treatment durations. Such differences may reflect labour market structures that decrease manual workers’ possibilities to engage and attend regular treatment sessions when compared with higher-level employees. Research has suggested that individuals with lower levels of education and occupational status may face a combination of practical (e.g. schedules, transportation), psychological (e.g. sense of stigma) and cultural (e.g. divergent expectations for the purpose of treatment between the client and higher socioeconomic background therapist) barriers that can impact their engagement in mental health treatment (Krupnick & Melnikoff, 2012; Levy & O’Hara, 2010). The differences in treatment duration according to education or occupational status were, however, rather moderate and thus such mechanisms should not be overemphasized. Interestingly, we also observed that lower income was associated with slightly longer treatment durations, which was somewhat unexpected given the associations observed with education and occupational status. However, the effect between income and treatment duration was weak. It may be that other factors related to labour market structures and working alliance, as well as expectations of treatment (Sharf et al., 2010; Smith et al., 2011) play a larger role in mediating treatment duration, particularly in countries like Finland with relatively high social security.

Our findings indicate that individuals living in the more sparsely populated eastern and northern regions of Finland had slightly shorter treatment durations compared to those residing in the more densely populated capital region. This effect was particularly pronounced among the most recent cohort. Previous research has suggested that greater distances to mental health treatment are associated with lower rates of treatment utilization (Finegan et al., 2020; Packness et al., 2017). Thus, it is likely that this disparity in treatment duration is related to differences in available treatment options and the distance that individuals must travel to access these services. Furthermore, it has been reported that psychotherapy treatment provision in Finland is primarily concentrated around university hospitals, except for the city of Tampere in southern Finland (Patana, 2014). This distribution of resources results in a scarcity of available treatment options in many parts of the country. Our findings indicate that this scarcity of resources and distance to treatment are associated with differences in treatment duration. Specifically, our results suggest that the disparity in treatment duration has increased between areas during the 2010s.

Our results suggest that the treatment duration has increased in the 2010s as individuals in the latter cohorts had the longest treatment durations. A legislative reform in 2011 made rehabilitative psychotherapy statutory, after which the number of its´ users has increased nearly threefold from the year 2011 to the year 2019 (Kela, 2022). Rapid growth has turned into a shortage of psychotherapists, challenges to find a therapist and several months or even a year of waiting time before the actual treatment begins. Longer time with functional impairment due to mental disorders has been associated with a longer total duration of the impairment after psychotherapy has begun (Alonso et al., 2018). Thus, our results raise a question of whether the longer waiting times for treatment have led to longer treatment durations. If so, actions to improve the client´s functioning should focus on reducing the waiting times for psychotherapy. However, our results also showed a significant variation in treatment durations, raising the question of whether all clients receiving rehabilitative psychotherapy require such long-term rehabilitation or whether another type of mental health treatment would be more suitable. This also points towards possible variations in treatment practices among different population groups. Prior research has highlighted the increased prevalence of mental health issues in lower socioeconomic status groups (Lorant et al., 2003). However, our findings suggest that these groups tend to receive shorter psychotherapy sessions. Moreover, as discussed earlier, men might be more inclined to discontinue treatment even when their symptoms have only slightly improved, while women persist with treatment despite symptom alleviation. Additionally, women generally exhibit more favorable attitudes toward mental health treatment. Yet, there is evidence suggesting gender bias in diagnosis and psychotropic drug treatment, with women being more likely to receive such treatment regardless of their health status or frequency of health service utilization compared to men (Bacigalupe & Martín, 2021). Our results may indicate similar attitudinal disparities in treatment implementation across various population segments. If this is the case, it is essential to consider the possibility of both under- and overtreatment, aspects that have received limited attention in psychotherapy research. Although our study’s design doesn’t allow us to directly explore these possibilities, the results highlight systematic differences that warrant further investigation. This is crucial to prevent potential harm arising from varying treatment patterns.

A strength of this study is its large population-based administrative register data, which represents psychotherapy use among 33% random samples of the working age population in three different cohorts. The dataset allowed the examination of the association of a comprehensive set of sociodemographic factors with treatment duration. Although the effects of single socioeconomic factors on treatment duration were moderate, the intersection of several factors contributed to considerable differences between the shortest and the longest treatment durations. Such results suggest that using only a single predictor might diminish the impact of an individual´s social background on treatment use. Additionally, the follow-up was relatively long allowing us to assess the change in treatment duration over time and the change in the magnitude of single factors on treatment duration. Thus far population-based studies have mostly examined whether sociodemographic factors associated with treatment use but not treatment duration (Boerema et al., 2017; Epping et al., 2017; Jokela et al., 2013b; Leppänen et al., 2022). On the other hand, studies that have investigated psychotherapy duration have typically focused on specific patient groups or types of psychotherapy (Lutz et al., 2015; Perry et al., 2007; Thompson et al., 2018). Consequently, previous research has not accounted for the variability in treatment duration within a population with equal treatment eligibility criteria. Our study addresses this gap and provides insight into the association between psychotherapy implementation and social determinants in the population.

This study has some limitations. First, we could not address the issue of unmet need because we did not have information on the clinical severity of the study population´s mental disorders. However, the criteria for rehabilitative psychotherapy are the same for everyone in Finland which supports the assumption that the clinical condition and the functional impairment due to the disorder were somewhat similar among the study population at the baseline assessment period. Second, despite the comprehensive set of sociodemographic measurements, they were rather traditional and may not necessarily capture how individuals interpret and express their social background (Liu et al., 2004). For example, engaging in treatment may be mediated by an individual´s worldview, such as their understanding of their possibilities and abilities, which could impact their help-seeking behavior. Moreover, this study was conducted among a rather ethnically homogenous Finnish population, and further research is needed to assess client social background-related factors that contribute to treatment duration. Although our sample size is large, the results of the interaction analysis should be interpreted with caution, as the analysis might have lacked sufficient statistical power to detect interactions of small magnitude. Our sensitivity analyses indicated some differences in psychotherapy utilization patterns between population groups in our earliest cohort (2011) compared with more recent cohorts (2013 and 2016). There are some indications that low income was linked to a longer treatment duration in the earliest cohort, but this relationship did not hold in the more recent cohorts. These findings suggest that there may have been variations in psychotherapy utilization tendencies among those who initiated treatment shortly after the reimbursement reform. While utilization rates increased in later cohorts, these differences underscore the potential influence of legislative reform on psychotherapy utilization patterns, particularly in the earliest cohort. As such, some caution should be exercised when considering the results regarding the earliest cohort. Finally, the follow-up for the earliest cohort (2010) was longer than for the more recent cohorts (2013 and 2016), which meant that overall durations exceeding 7 (in the 2013 cohort) or 4 (in the 2016 cohort) years could not be observed. Nevertheless, our results support the assumption that the overall treatment duration has increased despite the shorter follow-up period, which gives us confidence in the results.

We lacked detailed data on treatment implementation, including whether psychotherapists and clients had agreed on a specific treatment duration, or if the duration was more open-ended or based on the maximum number of approved sessions by the Social Insurance Institution of Finland. We observed that treatments often ended near the end of the first, second, or third year, suggesting that the approved yearly period strongly influenced the duration. However, some treatments ended at other points during the year-long periods. The register data did not provide information on treatment dropouts, changes of therapists during rehabilitation, or prior agreements between clients and therapists regarding the treatment duration. Therefore, we could not assess whether the duration was influenced by a client’s fast recovery, unexpected events during treatment (such as alliance ruptures), or other factors. Further research is necessary to examine more detailed factors that influence treatment duration and whether they differ systematically between population groups.

Finally, evidence suggests that longer treatment duration might prevent the need for new mental health treatment (Boerema et al., 2016) and that more frequent sessions can lead to faster improvement (Erekson et al., 2015; Robinson et al., 2020). However, since this study suggests that treatment implementation is associated with a client’s social background and area of residence, it would be of future interest to investigate whether these factors also moderate treatment outcomes in the population.

## Conclusions

This population-based study shows that despite equal clinical criteria for rehabilitative psychotherapy, individuals with different social backgrounds and residing in different areas systematically have different durations of treatment. The study also found that the average treatment duration increased during the 2010s, suggesting a widening inequality in treatment provision. Overall, the variation in the length of rehabilitative psychotherapy among the working population provides a new perspective on the inequalities in mental health care.

### Electronic Supplementary Material

Below is the link to the electronic supplementary material.

Supplementary Material 1

## Data Availability

Data in this study were used under its´ license approved by Statistics Finland, the Social Security Institution of Finland, Finnish Centre for Pensions. They are not publicly available. Currently, the Finnish Social and Health Data Permit Authority (Findata) coordinates the permissions for similar datasets, and they are available to researchers under permission (https://findata.fi/en/).

## Footnotes

1 Before 2011, receiving rehabilitative psychotherapy was based on a discretionary decision by the Social Insurance Institution of Finland and the annual budget limited its´ use. In 2011 it was made available for all that fulfill the criteria.
