# Supplementary material for: Sociodemographic Factors as Predictors of the Duration of Long-term Psychotherapy: Evidence from a Finnish Nationwide Register Study
Source: Adm Policy Ment Health. 2023 Oct 12;51(1):35–46. doi: 10.1007/s10488-023-01305-7 (PMC10791957; doi:10.1007/s10488-023-01305-7)
Supplement: Supplementary file 1 — Supplementary Material 1 [file 10488_2023_1305_MOESM1_ESM.docx]

Supplemental material for the original paper entitled *Sociodemographic factors as predictors of the duration of long-term psychotherapy: evidence from a Finnish nationwide register study* by Sanna Selinheimo, Kia Gluschkoff, Johanna Kausto, Jarno Turunen and Ari Väänänen

| Supplemental Table 1. Predicted treatment duration between combinations of sociodemographic factors in cohort 2013 | | | | | | | | | |  |
| --- | --- | --- | --- | --- | --- | --- | --- | --- | --- | --- |
| Duration, predicted | 95% CI (low, high) | |  |  |  | Occupational status |  | Area of residence |  |  |
|  |  |  | Age | Sex | Education |  | Income |  |  |  |
| 20.7 | 19.7 | 21.7 | 50 | M | Low | Manual workers | 4th (wealthiest) | East and North |  |  |
| 21.7 | 20.5 | 22.9 | 50 | M | Low | Manual workers | 4th (wealthiest) | Capital |  |  |
| 21.7 | 20.8 | 22.6 | 50 | M | Low | Manual workers | 1st (poorest) | East and North |  |  |
| 21.8 | 20.7 | 23.0 | 50 | M | Low | Upper-level | 4th (wealthiest) | East and North |  |  |
| 21.8 | 20.7 | 23.1 | 30 | M | Low | Manual workers | 4th (wealthiest) | East and North |  |  |
| 22.3 | 20.9 | 23.7 | 50 | F | Low | Manual workers | 4th (wealthiest) | East and North |  |  |
| 22.7 | 20.9 | 24.5 | 50 | M | Low | Manual workers | 1st (poorest) | Capital |  |  |
| 22.8 | 21.8 | 23.9 | 50 | M | Low | Upper-level | 4th (wealthiest) | Capital |  |  |
| 22.8 | 21.8 | 23.9 | 50 | M | Low | Upper-level | 1st (poorest) | East and North |  |  |
| 22.8 | 21.8 | 23.9 | 50 | M | High | Manual workers | 4th (wealthiest) | East and North |  |  |
| 22.9 | 21.8 | 23.9 | 30 | M | Low | Manual workers | 4th (wealthiest) | Capital |  |  |
| 22.9 | 21.8 | 24.0 | 30 | M | Low | Manual workers | 1st (poorest) | East and North |  |  |
| 23.0 | 21.9 | 24.2 | 30 | M | Low | Upper-level | 4th (wealthiest) | East and North |  |  |
| 23.3 | 22.3 | 24.4 | 50 | F | Low | Manual workers | 4th (wealthiest) | Capital |  |  |
| 23.3 | 22.3 | 24.4 | 50 | F | Low | Manual workers | 1st (poorest) | East and North |  |  |
| 23.5 | 22.5 | 24.5 | 50 | F | Low | Upper-level | 4th (wealthiest) | East and North |  |  |
| 23.5 | 22.2 | 25.0 | 30 | F | Low | Manual workers | 4th (wealthiest) | East and North |  |  |
| 23.9 | 22.8 | 25.0 | 50 | M | Low | Upper-level | 1st (poorest) | Capital |  |  |
| 23.9 | 22.8 | 25.1 | 50 | M | High | Manual workers | 4th (wealthiest) | Capital |  |  |
| 23.9 | 22.7 | 25.1 | 50 | M | High | Manual workers | 1st (poorest) | East and North |  |  |
| 23.9 | 22.8 | 25.2 | 30 | M | Low | Manual workers | 1st (poorest) | Capital |  |  |
| 24.1 | 23.0 | 25.2 | 50 | M | High | Upper-level | 4th (wealthiest) | East and North |  |  |
| 24.1 | 23.1 | 25.1 | 30 | M | Low | Upper-level | 4th (wealthiest) | Capital |  |  |
| 24.1 | 22.8 | 25.4 | 30 | M | Low | Upper-level | 1st (poorest) | East and North |  |  |
| 24.1 | 22.9 | 25.4 | 30 | M | High | Manual workers | 4th (wealthiest) | East and North |  |  |
| 24.4 | 23.2 | 25.6 | 50 | F | Low | Manual workers | 1st (poorest) | Capital |  |  |
| 24.6 | 23.3 | 26.0 | 50 | F | Low | Upper-level | 4th (wealthiest) | Capital |  |  |
| 24.6 | 23.6 | 25.6 | 50 | F | Low | Upper-level | 1st (poorest) | East and North |  |  |
| 24.6 | 23.4 | 25.9 | 50 | F | High | Manual workers | 4th (wealthiest) | East and North |  |  |
| 24.6 | 22.8 | 26.6 | 30 | F | Low | Manual workers | 4th (wealthiest) | Capital |  |  |
| 24.6 | 23.2 | 26.1 | 30 | F | Low | Manual workers | 1st (poorest) | East and North |  |  |
| 24.8 | 23.7 | 26.0 | 30 | F | Low | Upper-level | 4th (wealthiest) | East and North |  |  |
| 25.0 | 23.7 | 26.4 | 50 | M | High | Manual workers | 1st (poorest) | Capital |  |  |
| 25.2 | 23.8 | 26.7 | 50 | M | High | Upper-level | 4th (wealthiest) | Capital |  |  |
| 25.2 | 23.9 | 26.6 | 50 | M | High | Upper-level | 1st (poorest) | East and North |  |  |
| 25.2 | 24.1 | 26.4 | 30 | M | Low | Upper-level | 1st (poorest) | Capital |  |  |
| 25.2 | 24.2 | 26.3 | 30 | M | High | Manual workers | 4th (wealthiest) | Capital |  |  |
| 25.2 | 24.0 | 26.5 | 30 | M | High | Manual workers | 1st (poorest) | East and North |  |  |
| 25.4 | 24.1 | 26.8 | 30 | M | High | Upper-level | 4th (wealthiest) | East and North |  |  |
| 25.7 | 24.6 | 27.0 | 50 | F | Low | Upper-level | 1st (poorest) | Capital |  |  |
| 25.7 | 24.5 | 27.0 | 50 | F | High | Manual workers | 4th (wealthiest) | Capital |  |  |
| 25.7 | 24.6 | 26.9 | 50 | F | High | Manual workers | 1st (poorest) | East and North |  |  |
| 25.8 | 24.6 | 27.0 | 30 | F | Low | Manual workers | 1st (poorest) | Capital |  |  |
| 25.9 | 24.8 | 27.1 | 50 | F | High | Upper-level | 4th (wealthiest) | East and North |  |  |
| 26.0 | 24.7 | 27.3 | 30 | F | Low | Upper-level | 4th (wealthiest) | Capital |  |  |
| 26.0 | 24.7 | 27.3 | 30 | F | Low | Upper-level | 1st (poorest) | East and North |  |  |
| 26.0 | 24.6 | 27.4 | 30 | F | High | Manual workers | 4th (wealthiest) | East and North |  |  |
| 26.4 | 25.0 | 27.8 | 50 | M | High | Upper-level | 1st (poorest) | Capital |  |  |
| 26.4 | 25.2 | 27.6 | 30 | M | High | Manual workers | 1st (poorest) | Capital |  |  |
| 26.6 | 25.3 | 27.9 | 30 | M | High | Upper-level | 4th (wealthiest) | Capital |  |  |
| 26.6 | 25.5 | 27.7 | 30 | M | High | Upper-level | 1st (poorest) | East and North |  |  |
| 26.9 | 25.5 | 28.5 | 50 | F | High | Manual workers | 1st (poorest) | Capital |  |  |
| 27.1 | 26.0 | 28.3 | 50 | F | High | Upper-level | 4th (wealthiest) | Capital |  |  |
| 27.1 | 26.0 | 28.4 | 50 | F | High | Upper-level | 1st (poorest) | East and North |  |  |
| 27.2 | 25.8 | 28.7 | 30 | F | Low | Upper-level | 1st (poorest) | Capital |  |  |
| 27.2 | 26.0 | 28.4 | 30 | F | High | Manual workers | 4th (wealthiest) | Capital |  |  |
| 27.2 | 25.5 | 28.9 | 30 | F | High | Manual workers | 1st (poorest) | East and North |  |  |
| 27.4 | 26.2 | 28.6 | 30 | F | High | Upper-level | 4th (wealthiest) | East and North |  |  |
| 27.8 | 26.5 | 29.2 | 30 | M | High | Upper-level | 1st (poorest) | Capital |  |  |
| 28.4 | 27.2 | 29.6 | 50 | F | High | Upper-level | 1st (poorest) | Capital |  |  |
| 28.4 | 27.2 | 29.8 | 30 | F | High | Manual workers | 1st (poorest) | Capital |  |  |
| 28.7 | 27.4 | 30.0 | 30 | F | High | Upper-level | 4th (wealthiest) | Capital |  |  |
| 28.7 | 27.5 | 29.9 | 30 | F | High | Upper-level | 1st (poorest) | East and North |  |  |
| 30.0 | 28.4 | 31.6 | 30 | F | High | Upper-level | 1st (poorest) | Capital |  |  |
| CI = Confidence interval; Duration, predicted = Duration in months; F = Female; M = Male; Upper-level = Upper-level employees; 4th = 4th quartile; 1st = 1st quartile; Capital = Helsinki and Uusimaa | | | | | | | | | |  |
|  |  |  |  |  |  |  |  |  |  |  |

| Supplemental Table 2. Predicted treatment duration between combinations of sociodemographic factors in cohort 2013 | | | | | | | | |  |
| --- | --- | --- | --- | --- | --- | --- | --- | --- | --- |
| Duration, predicted | 95% CI (low, high) | |  |  |  | Occupational status |  | Area of residence |  |
|  |  |  | Age | Sex | Education |  | Income |  |  |
| 21.55 | 20.50 | 22.65 | 50 | M | Low | Manual workers | 4th (wealthiest) | East and North |  |
| 22.55 | 21.37 | 23.80 | 50 | M | Low | Manual workers | 4th (wealthiest) | Capital |  |
| 22.55 | 21.62 | 23.53 | 50 | M | Low | Manual workers | 1st (poorest) | East and North |  |
| 22.72 | 21.58 | 23.91 | 50 | M | Low | Upper-level | 4th (wealthiest) | East and North |  |
| 22.75 | 21.55 | 24.03 | 30 | M | Low | Manual workers | 4th (wealthiest) | East and North |  |
| 23.21 | 21.82 | 24.70 | 50 | F | Low | Manual workers | 4th (wealthiest) | East and North |  |
| 23.60 | 21.79 | 25.56 | 50 | M | Low | Manual workers | 1st (poorest) | Capital |  |
| 23.77 | 22.71 | 24.88 | 50 | M | Low | Upper-level | 4th (wealthiest) | Capital |  |
| 23.78 | 22.74 | 24.85 | 50 | M | Low | Upper-level | 1st (poorest) | East and North |  |
| 23.78 | 22.69 | 24.93 | 50 | M | High | Manual workers | 4th (wealthiest) | East and North |  |
| 23.81 | 22.74 | 24.93 | 30 | M | Low | Manual workers | 4th (wealthiest) | Capital |  |
| 23.81 | 22.72 | 24.96 | 30 | M | Low | Manual workers | 1st (poorest) | East and North |  |
| 23.99 | 22.82 | 25.21 | 30 | M | Low | Upper-level | 4th (wealthiest) | East and North |  |
| 24.29 | 23.26 | 25.37 | 50 | F | Low | Manual workers | 4th (wealthiest) | Capital |  |
| 24.29 | 23.23 | 25.41 | 50 | F | Low | Manual workers | 1st (poorest) | East and North |  |
| 24.47 | 23.44 | 25.55 | 50 | F | Low | Upper-level | 4th (wealthiest) | East and North |  |
| 24.51 | 23.09 | 26.02 | 30 | F | Low | Manual workers | 4th (wealthiest) | East and North |  |
| 24.88 | 23.74 | 26.08 | 50 | M | Low | Upper-level | 1st (poorest) | Capital |  |
| 24.89 | 23.70 | 26.13 | 50 | M | High | Manual workers | 4th (wealthiest) | Capital |  |
| 24.89 | 23.67 | 26.17 | 50 | M | High | Manual workers | 1st (poorest) | East and North |  |
| 24.92 | 23.70 | 26.20 | 30 | M | Low | Manual workers | 1st (poorest) | Capital |  |
| 25.07 | 23.97 | 26.22 | 50 | M | High | Upper-level | 4th (wealthiest) | East and North |  |
| 25.10 | 24.06 | 26.18 | 30 | M | Low | Upper-level | 4th (wealthiest) | Capital |  |
| 25.10 | 23.78 | 26.50 | 30 | M | Low | Upper-level | 1st (poorest) | East and North |  |
| 25.11 | 23.84 | 26.44 | 30 | M | High | Manual workers | 4th (wealthiest) | East and North |  |
| 25.42 | 24.20 | 26.71 | 50 | F | Low | Manual workers | 1st (poorest) | Capital |  |
| 25.61 | 24.22 | 27.07 | 50 | F | Low | Upper-level | 4th (wealthiest) | Capital |  |
| 25.61 | 24.58 | 26.68 | 50 | F | Low | Upper-level | 1st (poorest) | East and North |  |
| 25.62 | 24.38 | 26.92 | 50 | F | High | Manual workers | 4th (wealthiest) | East and North |  |
| 25.65 | 23.70 | 27.75 | 30 | F | Low | Manual workers | 4th (wealthiest) | Capital |  |
| 25.65 | 24.17 | 27.23 | 30 | F | Low | Manual workers | 1st (poorest) | East and North |  |
| 25.84 | 24.65 | 27.08 | 30 | F | Low | Upper-level | 4th (wealthiest) | East and North |  |
| 26.04 | 24.68 | 27.49 | 50 | M | High | Manual workers | 1st (poorest) | Capital |  |
| 26.23 | 24.75 | 27.81 | 50 | M | High | Upper-level | 4th (wealthiest) | Capital |  |
| 26.24 | 24.86 | 27.69 | 50 | M | High | Upper-level | 1st (poorest) | East and North |  |
| 26.27 | 25.06 | 27.53 | 30 | M | Low | Upper-level | 1st (poorest) | Capital |  |
| 26.27 | 25.16 | 27.44 | 30 | M | High | Manual workers | 4th (wealthiest) | Capital |  |
| 26.28 | 25.02 | 27.60 | 30 | M | High | Manual workers | 1st (poorest) | East and North |  |
| 26.47 | 25.11 | 27.91 | 30 | M | High | Upper-level | 4th (wealthiest) | East and North |  |
| 26.80 | 25.59 | 28.07 | 50 | F | Low | Upper-level | 1st (poorest) | Capital |  |
| 26.81 | 25.56 | 28.11 | 50 | F | High | Manual workers | 4th (wealthiest) | Capital |  |
| 26.81 | 25.63 | 28.04 | 50 | F | High | Manual workers | 1st (poorest) | East and North |  |
| 26.84 | 25.59 | 28.16 | 30 | F | Low | Manual workers | 1st (poorest) | Capital |  |
| 27.01 | 25.81 | 28.25 | 50 | F | High | Upper-level | 4th (wealthiest) | East and North |  |
| 27.04 | 25.74 | 28.41 | 30 | F | Low | Upper-level | 4th (wealthiest) | Capital |  |
| 27.04 | 25.72 | 28.43 | 30 | F | Low | Upper-level | 1st (poorest) | East and North |  |
| 27.05 | 25.60 | 28.58 | 30 | F | High | Manual workers | 4th (wealthiest) | East and North |  |
| 27.46 | 26.05 | 28.94 | 50 | M | High | Upper-level | 1st (poorest) | Capital |  |
| 27.50 | 26.26 | 28.79 | 30 | M | High | Manual workers | 1st (poorest) | Capital |  |
| 27.70 | 26.36 | 29.10 | 30 | M | High | Upper-level | 4th (wealthiest) | Capital |  |
| 27.70 | 26.57 | 28.88 | 30 | M | High | Upper-level | 1st (poorest) | East and North |  |
| 28.05 | 26.51 | 29.69 | 50 | F | High | Manual workers | 1st (poorest) | Capital |  |
| 28.26 | 27.08 | 29.49 | 50 | F | High | Upper-level | 4th (wealthiest) | Capital |  |
| 28.26 | 27.04 | 29.54 | 50 | F | High | Upper-level | 1st (poorest) | East and North |  |
| 28.30 | 26.83 | 29.85 | 30 | F | Low | Upper-level | 1st (poorest) | Capital |  |
| 28.30 | 27.06 | 29.61 | 30 | F | High | Manual workers | 4th (wealthiest) | Capital |  |
| 28.31 | 26.60 | 30.12 | 30 | F | High | Manual workers | 1st (poorest) | East and North |  |
| 28.51 | 27.34 | 29.74 | 30 | F | High | Upper-level | 4th (wealthiest) | East and North |  |
| 28.99 | 27.60 | 30.45 | 30 | M | High | Upper-level | 1st (poorest) | Capital |  |
| 29.58 | 28.35 | 30.86 | 50 | F | High | Upper-level | 1st (poorest) | Capital |  |
| 29.62 | 28.28 | 31.02 | 30 | F | High | Manual workers | 1st (poorest) | Capital |  |
| 29.84 | 28.50 | 31.24 | 30 | F | High | Upper-level | 4th (wealthiest) | Capital |  |
| 29.84 | 28.61 | 31.13 | 30 | F | High | Upper-level | 1st (poorest) | East and North |  |
| 31.23 | 29.62 | 32.92 | 30 | F | High | Upper-level | 1st (poorest) | Capital |  |
| CI = Confidence interval; Duration, predicted = Duration in months; F = Female; M = Male; Upper-level = Upper-level employees; 4th = 4th quartile; 1st = 1st quartile; Capital = Helsinki and Uusimaa | | | | | | | | |  |
|  |  |  |  |  |  |  |  |  |  |

| Supplemental Table 3. Predicted treatment duration between combinations of sociodemographic factors in cohort 2016 | | | | | | | | |  |
| --- | --- | --- | --- | --- | --- | --- | --- | --- | --- |
| Duration, predicted | 95% CI (low, high) | |  |  |  |  |  |  |  |
|  |  |  | Age | Sex | Education | Occupational status | Income | Area of residence |  |
| 21.9 | 20.9 | 23.0 | 50 | M | Low | Manual workers | 4th (wealthiest) | East and North |  |
| 22.9 | 21.7 | 24.2 | 50 | M | Low | Manual workers | 4th (wealthiest) | Capital |  |
| 22.9 | 22.0 | 23.9 | 50 | M | Low | Manual workers | 1st (poorest) | East and North |  |
| 23.1 | 22.0 | 24.3 | 50 | M | Low | Upper-level | 4th (wealthiest) | East and North |  |
| 23.1 | 21.9 | 24.4 | 30 | M | Low | Manual workers | 4th (wealthiest) | East and North |  |
| 23.6 | 22.2 | 25.1 | 50 | F | Low | Manual workers | 4th (wealthiest) | East and North |  |
| 24.0 | 22.2 | 26.0 | 50 | M | Low | Manual workers | 1st (poorest) | Capital |  |
| 24.2 | 23.1 | 25.3 | 50 | M | Low | Upper-level | 4th (wealthiest) | Capital |  |
| 24.2 | 23.1 | 25.3 | 50 | M | Low | Upper-level | 1st (poorest) | East and North |  |
| 24.2 | 23.1 | 25.4 | 50 | M | High | Manual workers | 4th (wealthiest) | East and North |  |
| 24.2 | 23.1 | 25.4 | 30 | M | Low | Manual workers | 4th (wealthiest) | Capital |  |
| 24.2 | 23.1 | 25.4 | 30 | M | Low | Manual workers | 1st (poorest) | East and North |  |
| 24.4 | 23.2 | 25.7 | 30 | M | Low | Upper-level | 4th (wealthiest) | East and North |  |
| 24.7 | 23.7 | 25.8 | 50 | F | Low | Manual workers | 4th (wealthiest) | Capital |  |
| 24.7 | 23.6 | 25.9 | 50 | F | Low | Manual workers | 1st (poorest) | East and North |  |
| 24.9 | 23.8 | 26.0 | 50 | F | Low | Upper-level | 4th (wealthiest) | East and North |  |
| 24.9 | 23.5 | 26.5 | 30 | F | Low | Manual workers | 4th (wealthiest) | East and North |  |
| 25.3 | 24.2 | 26.5 | 50 | M | Low | Upper-level | 1st (poorest) | Capital |  |
| 25.3 | 24.1 | 26.6 | 50 | M | High | Manual workers | 4th (wealthiest) | Capital |  |
| 25.3 | 24.1 | 26.6 | 50 | M | High | Manual workers | 1st (poorest) | East and North |  |
| 25.4 | 24.1 | 26.7 | 30 | M | Low | Manual workers | 1st (poorest) | Capital |  |
| 25.5 | 24.4 | 26.7 | 50 | M | High | Upper-level | 4th (wealthiest) | East and North |  |
| 25.5 | 24.5 | 26.6 | 30 | M | Low | Upper-level | 4th (wealthiest) | Capital |  |
| 25.5 | 24.2 | 27.0 | 30 | M | Low | Upper-level | 1st (poorest) | East and North |  |
| 25.5 | 24.3 | 26.9 | 30 | M | High | Manual workers | 4th (wealthiest) | East and North |  |
| 25.9 | 24.6 | 27.2 | 50 | F | Low | Manual workers | 1st (poorest) | Capital |  |
| 26.1 | 24.6 | 27.5 | 50 | F | Low | Upper-level | 4th (wealthiest) | Capital |  |
| 26.1 | 25.0 | 27.1 | 50 | F | Low | Upper-level | 1st (poorest) | East and North |  |
| 26.1 | 24.8 | 27.4 | 50 | F | High | Manual workers | 4th (wealthiest) | East and North |  |
| 26.1 | 24.1 | 28.2 | 30 | F | Low | Manual workers | 4th (wealthiest) | Capital |  |
| 26.1 | 24.6 | 27.7 | 30 | F | Low | Manual workers | 1st (poorest) | East and North |  |
| 26.3 | 25.1 | 27.6 | 30 | F | Low | Upper-level | 4th (wealthiest) | East and North |  |
| 26.5 | 25.1 | 28.0 | 50 | M | High | Manual workers | 1st (poorest) | Capital |  |
| 26.7 | 25.2 | 28.3 | 50 | M | High | Upper-level | 4th (wealthiest) | Capital |  |
| 26.7 | 25.3 | 28.2 | 50 | M | High | Upper-level | 1st (poorest) | East and North |  |
| 26.7 | 25.5 | 28.0 | 30 | M | Low | Upper-level | 1st (poorest) | Capital |  |
| 26.7 | 25.6 | 27.9 | 30 | M | High | Manual workers | 4th (wealthiest) | Capital |  |
| 26.7 | 25.5 | 28.1 | 30 | M | High | Manual workers | 1st (poorest) | East and North |  |
| 26.9 | 25.5 | 28.4 | 30 | M | High | Upper-level | 4th (wealthiest) | East and North |  |
| 27.3 | 26.0 | 28.6 | 50 | F | Low | Upper-level | 1st (poorest) | Capital |  |
| 27.3 | 26.0 | 28.6 | 50 | F | High | Manual workers | 4th (wealthiest) | Capital |  |
| 27.3 | 26.1 | 28.5 | 50 | F | High | Manual workers | 1st (poorest) | East and North |  |
| 27.3 | 26.0 | 28.6 | 30 | F | Low | Manual workers | 1st (poorest) | Capital |  |
| 27.5 | 26.3 | 28.7 | 50 | F | High | Upper-level | 4th (wealthiest) | East and North |  |
| 27.5 | 26.2 | 28.9 | 30 | F | Low | Upper-level | 4th (wealthiest) | Capital |  |
| 27.5 | 26.2 | 28.9 | 30 | F | Low | Upper-level | 1st (poorest) | East and North |  |
| 27.5 | 26.0 | 29.1 | 30 | F | High | Manual workers | 4th (wealthiest) | East and North |  |
| 27.9 | 26.5 | 29.4 | 50 | M | High | Upper-level | 1st (poorest) | Capital |  |
| 28.0 | 26.7 | 29.3 | 30 | M | High | Manual workers | 1st (poorest) | Capital |  |
| 28.2 | 26.8 | 29.6 | 30 | M | High | Upper-level | 4th (wealthiest) | Capital |  |
| 28.2 | 27.0 | 29.4 | 30 | M | High | Upper-level | 1st (poorest) | East and North |  |
| 28.5 | 27.0 | 30.2 | 50 | F | High | Manual workers | 1st (poorest) | Capital |  |
| 28.8 | 27.6 | 30.0 | 50 | F | High | Upper-level | 4th (wealthiest) | Capital |  |
| 28.8 | 27.5 | 30.1 | 50 | F | High | Upper-level | 1st (poorest) | East and North |  |
| 28.8 | 27.3 | 30.4 | 30 | F | Low | Upper-level | 1st (poorest) | Capital |  |
| 28.8 | 27.5 | 30.1 | 30 | F | High | Manual workers | 4th (wealthiest) | Capital |  |
| 28.8 | 27.1 | 30.6 | 30 | F | High | Manual workers | 1st (poorest) | East and North |  |
| 29.0 | 27.8 | 30.3 | 30 | F | High | Upper-level | 4th (wealthiest) | East and North |  |
| 29.5 | 28.1 | 31.0 | 30 | M | High | Upper-level | 1st (poorest) | Capital |  |
| 30.1 | 28.8 | 31.4 | 50 | F | High | Upper-level | 1st (poorest) | Capital |  |
| 30.1 | 28.8 | 31.6 | 30 | F | High | Manual workers | 1st (poorest) | Capital |  |
| 30.4 | 29.0 | 31.8 | 30 | F | High | Upper-level | 4th (wealthiest) | Capital |  |
| 30.4 | 29.1 | 31.7 | 30 | F | High | Upper-level | 1st (poorest) | East and North |  |
| 31.8 | 30.1 | 33.5 | 30 | F | High | Upper-level | 1st (poorest) | Capital |  |
| CI = Confidence interval; Duration, predicted = Duration in months; F = Female; M = Male; Upper-level = Upper-level employees; 4th = 4th quartile; 1st = 1st quartile; Capital = Helsinki and Uusimaa | | | | | | | | |  |
|  |  |  |  |  |  |  |  |  |  |


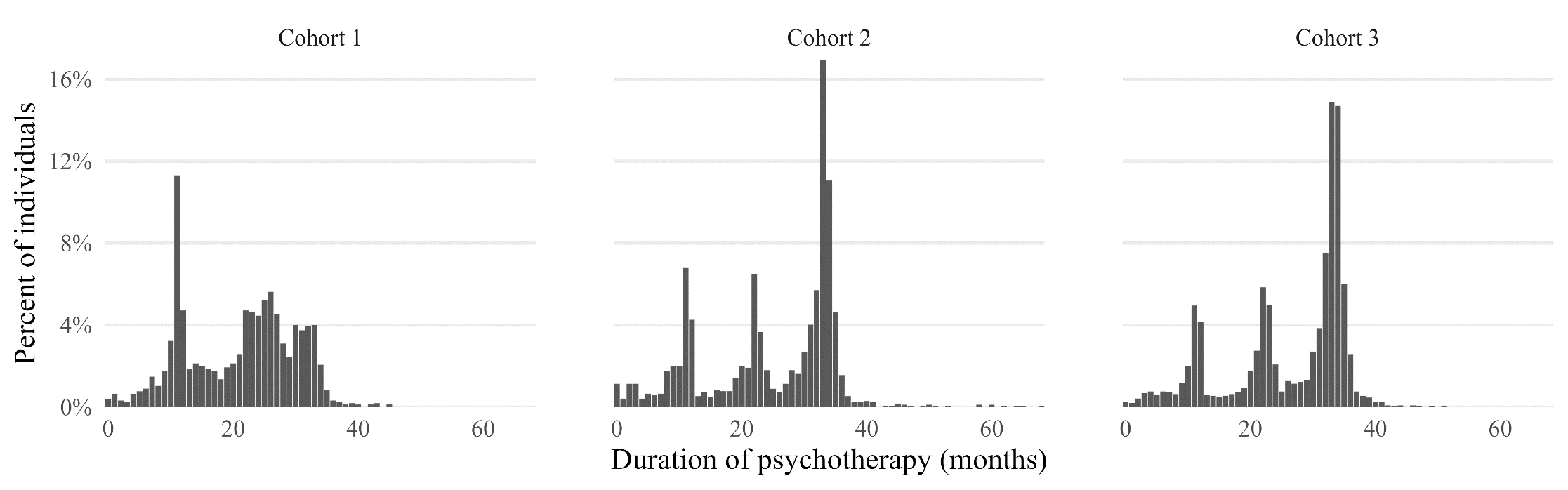


Supplemental Fig 1 Treatment duration in months (excluding breaks) in study cohorts.


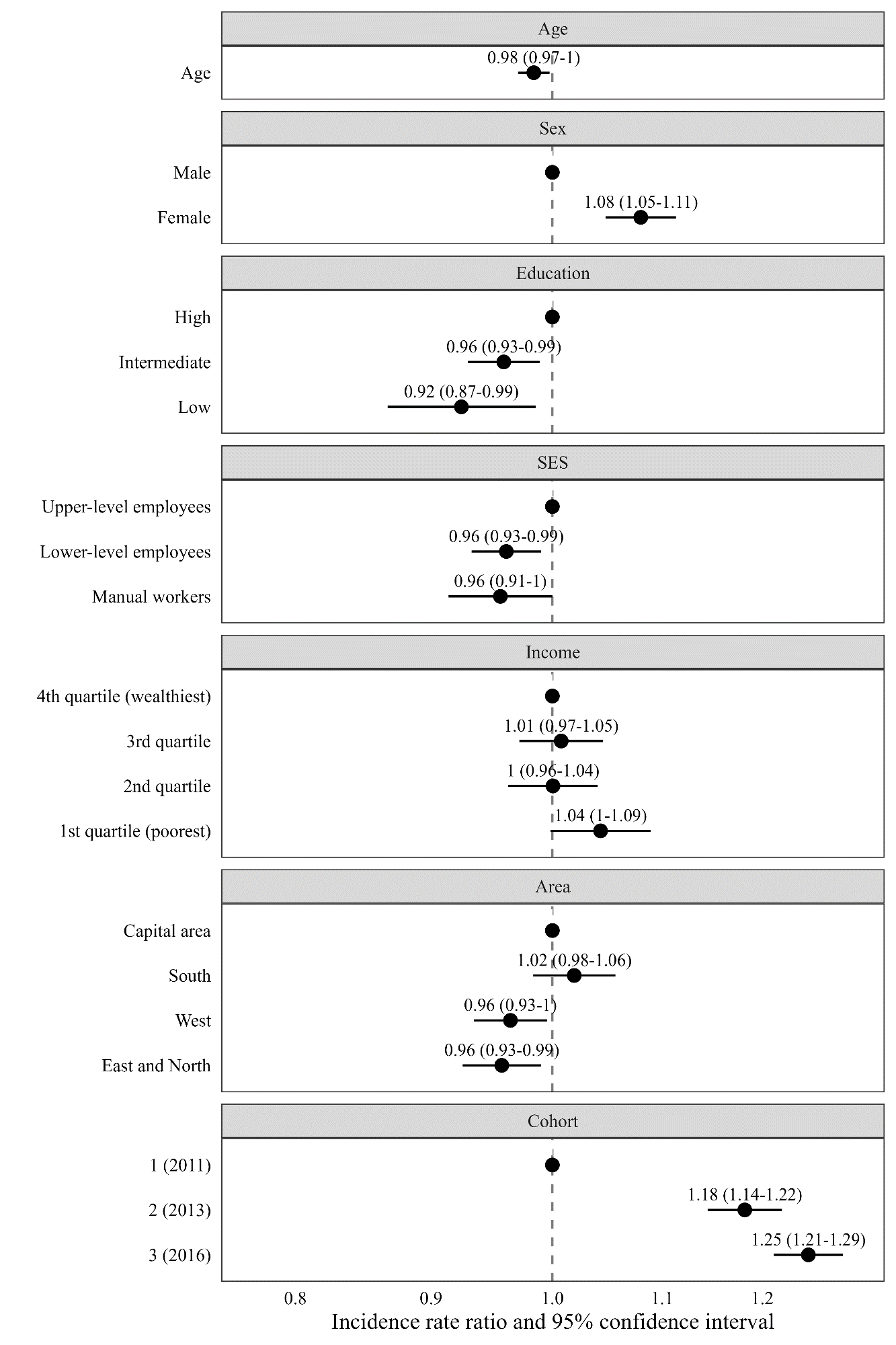


Supplemental Fig 2 Incidence rate ratios (95% confidence interval) for the associations between sociodemographic factors and psychotherapy duration. Age = The effect of a 10-year increase in age. Treatment duration was calculated by excluding breaks between treatment periods from the total duration.


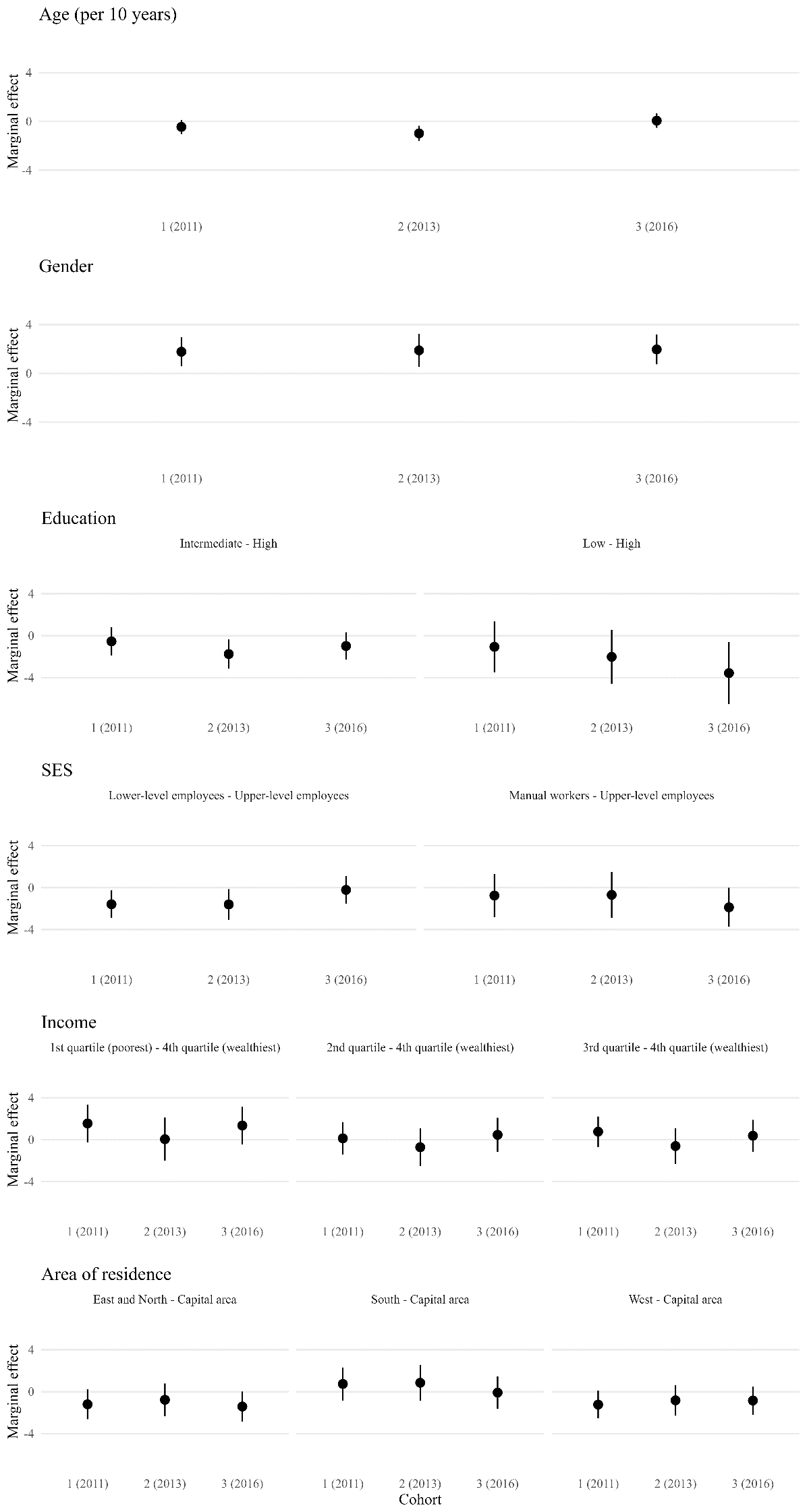


*Supplemental Fig 3 Marginal effects for predictors of treatment duration calculated by excluding breaks between treatment periods in the duration.*

*

*

*Supplemental Fig 4 Incidence rate ratios (95% confidence interval) for the associations between sociodemographic factors and psychotherapy duration separately for 1) cohort 1 and 2) cohorts 2 and 3. Psychotherapy duration in years (1, 2 or 3) was modelled with multinomial regression, with the duration of 1 year as the reference category. Age = The effect of a 10-year increase in age.*
